# A Case Report: An Elderly Male Patient With Takayasu Arteritis After Coronary Artery Bypass Grafting

**DOI:** 10.3389/fcvm.2021.766574

**Published:** 2021-11-23

**Authors:** Huaitao Yu, Wenzhao Liu, Yuan Zhang, Xuefang Yan, Na Li, Yun Ti, Peili Bu

**Affiliations:** Key Laboratory of Cardiovascular Remodeling and Function Research, Chinese Ministry of Education, Chinese National Health Commission and Chinese Academy of Medical Sciences, State and Shandong Joint Key Laboratory of Translational Cardiovascular Medicine, Department of Cardiology, Qilu Hospital, Cheeloo College of Medicine, Shandong University, Jinan, China

**Keywords:** case report, Takayasu's arteritis, coronary artery bypass grafting, Takayasu's arteritis with coronary artery involvement, acute myocardial infarction

## Abstract

**Background:** Takayasu arteritis is a rare chronic granulomatous inflammation involving the aorta and its main branches. In this report, we describe an extremely rare elderly male patient with Takayasu arteritis (TA) after coronary artery bypass grafting (CABG).

**Case Summary:** A 61-year-old male patient with persistent precordial pain underwent angiography. Vascular murmurs could be heard in carotid artery and bilateral renal artery by auscultation. Laboratory parameters showed high Erythrocyte Sedimentation Rate (ESR) and C-reactive protein (CRP). CT coronary angiography showed multiple stenoses of aorta and its main branches, such as carotid and renal artery involvement. Coronary angiography showed that the coronary artery had multiple branch stenoses, the left anterior descending artery (LAD) had severe stenosis, the distal end of which was reversed to the right coronary artery (RCA), and the RCA was completely occluded. Because of the high level of markers of inflammatory activity, the patient began to take glucocorticoid. Although the patient still had multibranch stenosis of coronary artery, considering the previous CABG operation history, surgery, and interventional therapy of the patient were not feasible, the patient was given conservative drug for further treatment. After treatment, the inflammatory index was significantly descended, and N terminal-pro Brain natriuretic peptide (NT-pro BNP) was decreased.

**Discussion:** A rare case of an elderly male patient with Takayasu arteritis after coronary artery bypass grafting was reported. In addition to hypertension, hyperlipidemia, and other risk factors, coronary artery involvement caused by TA may be a major cause of aggravation of symptoms in patients with acute myocardial infarction (AMI), especially after CABG.

## Introduction

Takayasu's arteritis is a chronic non-specific inflammation of artery, the case of which is rare. The lesion mainly involves the pulmonary artery and the aorta and its main branches. Most of the cases are female, and the age of the patients is among 20–40 (1). Sometimes, the coronary artery is invaded by the lesion, which we define as Takayasu's arteritis (TA) with coronary artery involvement. The cardiac involvement of TA can be classified as valvular heart disease, myocardial involvement, hypertensive heart disease, and coronary artery disease. The involvement of aortic valve can lead to regurgitation and heart failure. The damage of TA to the coronary artery can lead to coronary stenosis and occlusion, angina pectoris, and even acute myocardial infarction (2). Here, we present a case of an elderly male suffering from TA with coronary artery involvement. It is noteworthy that the patient was under coronary artery bypass grafting (CABG) before.

## Case Presentation

Our patient was a 61-year-old male. He was admitted into the cardiac care unit (CCU) of our hospital because of more than 16-h precordial pain. Sixteen hours ago, the precordial pain with no obvious cause occurred. The pain was persistent and accompanied by a radiation pain in left shoulder. There were no other concomitant symptoms, including chest tightness, panic, fever, nausea and vomiting, dizziness, headache, etc. There was no significant relief in the pain after taking “SUXIAO JIUXIN PILL.” Then, the patient called an ambulance and went to the local hospital. After the electrocardiogram examination, the local hospital considered the diagnosis as acute myocardial infarction, and oral doses of aspirin and clopidogrel were prescribed. For further diagnosis and treatment, the patient was transferred to the emergency department of our hospital. Electrocardiogram (ECG) examination showed that ST segment elevated in the lead of AVR and V1 and depressed in other leads. The laboratory examination showed that troponin was elevated, so he was admitted to the CCU of our hospital. Asking patients and his family members carefully, the patient underwent coronary angiography in our hospital 11 years ago, showing that 40% stenosis occurred in the distal segment of the left main coronary artery (LM); the stenosis of the proximal segment of LAD was 40%; long lesions in the proximal and middle left circumflex branch (LCX), the narrowest part of LCX was about 80%, and the distal segment of LCX was about 70%; nearly 70% stenosis occurred in the obtuse marginal branch 1 (OM1); the proximal segment of the RCA was completely occluded and the distal segment was visualized through the left coronary collateral branch. After evaluation, CABG was performed on him in our hospital. The patient had a history of hypertension for 11 years, and the blood pressure was as high as 180/110 mmHg. He took felodipine-sustained release tablets and irbesartan to control blood pressure and said that his blood pressure was well-controlled. He also had a history of diabetes for 11 years, and took Metformin and Acarbose for treatment, and his daily blood glucose was controlled at 7–8 mmol/L. He denied a history of chronic obstructive pulmonary disease (COPD), cerebrovascular disease, and other chronic diseases. The patient had 30 years of smoking history, about 20 cigarettes per day. At present, he had quit smoking for 11 years and drunk occasionally. Other family members had no similar clinical manifestations. Past medical history and family history revealed nothing significant.

On admission, his body temperature was 36.4°C, the pulse was 96 beats per minute, his respiratory rate was 18 times per minute, his blood pressure was 150/85 mmHg (1 mmHg = 0.133 kPa). Physical examination showed that the patient was in a poor state of mind and spirit. The patient had a mild coarse breath sounds on auscultation and obvious moist rales could be heard. The heart boundary (cardiac dullness) was not big. The heart rate was 96 beats per minute. The heart sounds were normal, and the rhythm was regular. There was no pathological murmur in each valve auscultation area. No edema was found in both lower limbs. There was no distension of jugular vein and no sign of hepatic jugular vein reflux.

**Figure 1:**
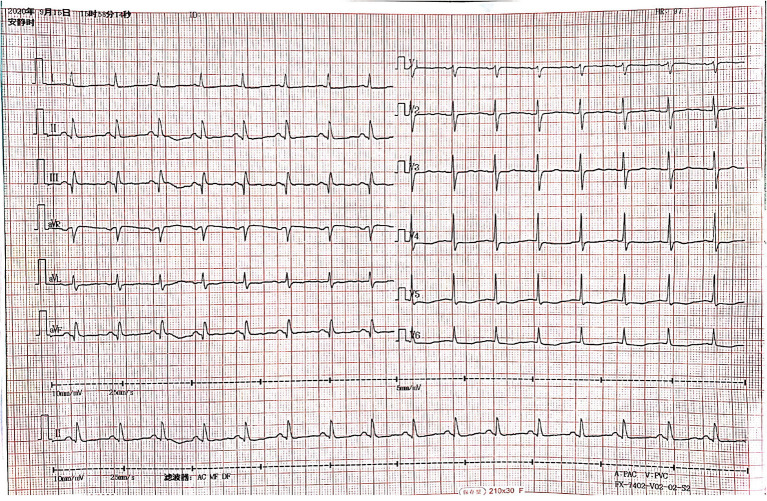
Electrocardiogram after admission to our hospital.

Laboratory examination: the results of myocardial enzyme spectrum showed that cardiac troponin I (cTnI) was 3.8 μg/L (reference value was 0–0.023 μg/L), creatine kinase isoenzyme (CK-MB) was 38 μg/L (reference value was 0–7.2 μg/L), NT-pro BNP was 5,530 pg/ml (reference value was 0–300 μg/L); liver function revealed an ALT level of 11 μ/L, an aspartate aminotransferase (AST) level of 28 μ/L, an albumin (ALB) level of 40 g/L; renal function showed an serum urea nitrogen (BUN) level of 5.2 mmol/L, a serum creatinine (Cr) level of 48 μmol/L; blood sugar (BS) was 9.37 mmol/L; blood biochemistry revealed a total cholesterol (TC) level of 3.7 mmol/L, a low-density lipoprotein (LDL-C) level of 2.98 mmol/L, a sodium (Na^+^) level of 133 mmol/L, a potassium (K^+^) level of 3.98 mmol/L. Blood routine showed that the white blood cell count (WBC) was 10.60 × 10^9^/L (reference value was 125–350 × 10^9^/L), the proportion of neutrophils (NEU%) was 81.40%; ESR was 106 mm/h; CRP was 107 mg/L; other examinations were in the normal range. The 12-lead electrocardiogram showed normal sinus rhythm, ST segment depression in lead V4–V6, T wave flattening or inversion in chest lead and limb lead, abnormal Q wave in lead III and AVF (Figure 1). Echocardiography revealed multiple abnormalities: (i) enlargement of the left heart, with a left atrium diameter of 45 mm and a left ventricular diameter of 48 mm; (ii) the inferior wall of left ventricle became thinner, and the motion almost disappeared; (iii) the activity of myocardium near the apex of interventricular septum decreased. The estimated left ventricular ejection fraction (LVEF) was 45%; (iv) moderate pulmonary hypertension; (v) mild-to-moderate mitral regurgitation, mild-to-moderate tricuspid regurgitation. No obvious abnormality of heart and lung was found in chest X-ray at the bedside.

Based on the main symptoms of the patient, typical ECG manifestations and changes of myocardial enzyme spectrum, and previous CABG operation history, the main diagnosis of the patient was acute non-ST segment elevation myocardial infarction (NSTEMI); the following diagnoses were made: (i) acute non-ST segment elevation myocardial infarction; (ii) hypertension (Grade 3, very high risk); (iii) Type 2 diabetes. Aspirin and clopidogrel were used to antiplatelet therapy, low molecular weight heparin (LMWH) was used to anti-coagulation, atorvastatin, and ezetimibe were used to decrease the serum lipid level. We used metoprolol, isosorbide mononitrate-sustained release tablets, and trimetazidine to relieve the symptom of angina pectoris; “metoprolol, valsartan, and amlodipine” were used to reduce blood pressure and improve ventricular remodeling; creatine phosphate was used to nourish the myocardium, nicorandil to improve microcirculation of coronary artery. Furosemide, spironolactone, and nesiritide can improve cardiac function, Dapagliptin, acarbose, and metformin were used to treat diabetes. After treatment, the condition of the patient was more stable than before. So, the patient underwent coronary angiography, showing that 50% stenosis occurred in the LM; the left anterior descending branch is small, with a stenosis of about 80% in the proximal segment and irregular in the middle and distal segments. The most severe stenosis is about 70%, and it can be seen that the distal part of the lad is retrograde to the right Corus artery; in LCX, the wall of proximal segment was irregular, the lesions in middle and distal segments were longer, and the most severe stenosis was about 90%; the wall of OM was irregular, and local stenosis was 40%; RCA was occluded from the proximal segment; the LCX bridge was unobstructed.

**Figure 2:**
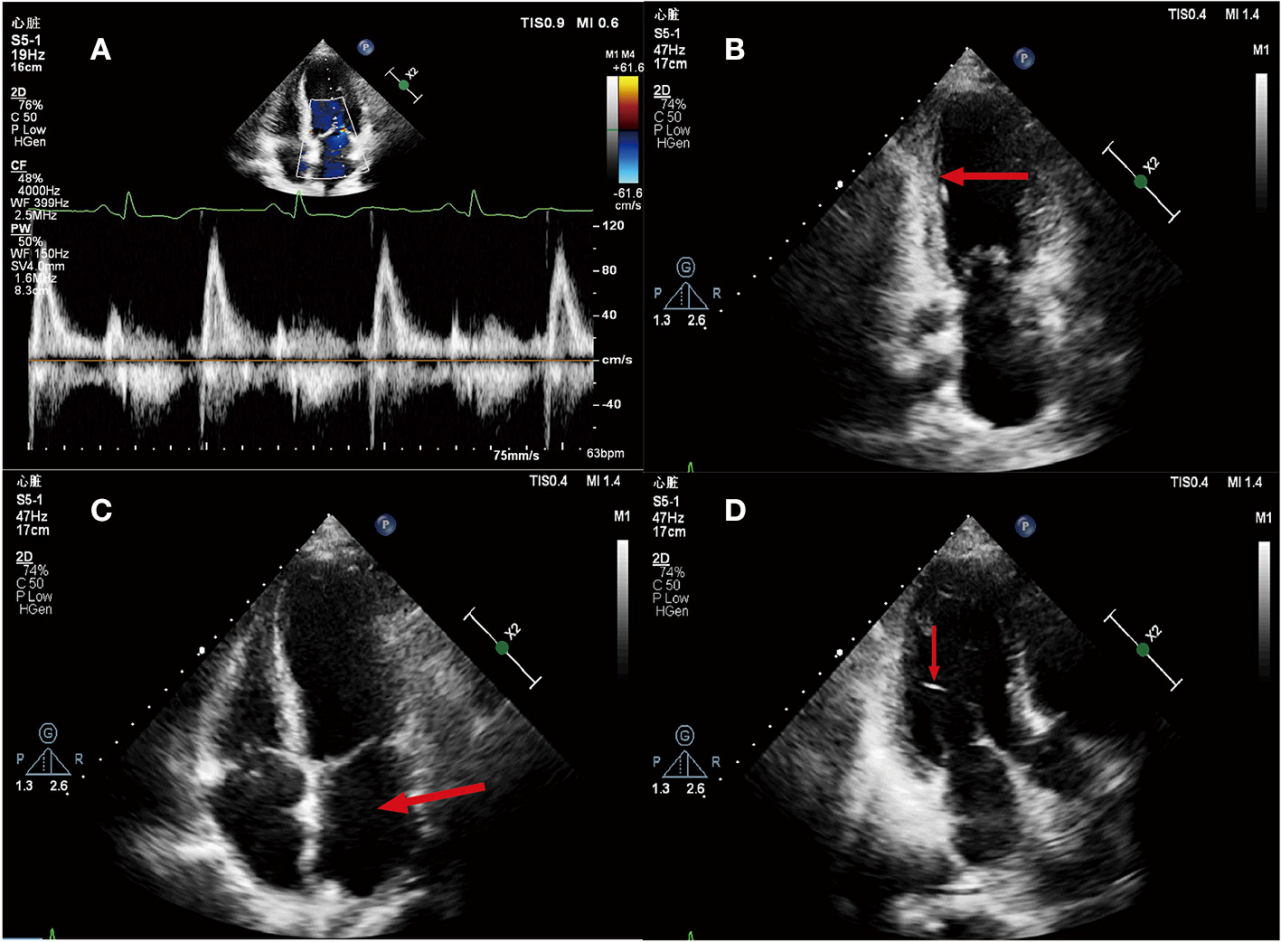
**(A)** Mitral flow spectrum Doppler; **(B)** Apical two-chamber heart view, Ventricular systole. The arrow shows that the inferior wall of left ventricle became thinner, the echo increased, and the mobility decreased; **(C)** Apical four-chamber heart view. The arrow indicates the enlargement of left atrial; **(D)** Apical two-chamber heart view, Ventricular systole. The arrow shows a left ventricular false tendon.

We reexamined the echocardiography of the patients and found: (i) it was in accordance with the echocardiographic findings of myocardial infarction; (ii) enlargement of the left heart, with a left atrium diameter of 41 mm and a left ventricular diameter of 45 mm; (iii) segmental dyskinesia of left ventricular wall and decreased left ventricular diastolic function was found. The LVEF was 51%; (iv) mild pulmonary hypertension; (v) mild mitral and tricuspid regurgitation (Figure 2).

The reexamination of cTnI was 0.01 μg/L (reference value was 0–0.023 μg/L), NT-pro BNP was 4,380 pg/ml (reference value was 0–300 μg/L); ESR was 109 mm/h; CRP was 107 mg/L. After the above positive treatment, the symptoms of precordial pain were significantly improved; the indexes related to myocardial enzyme spectrum were reduced to normal. The renal function and urine volume were acceptable. But the decrease of NT-pro BNP was not obvious; CRP and ESR were significantly increased during the treatment. We considered that pulmonary infection, tumor, and rheumatic disease could lead to significant increase of CRP and ESR. For this reason, we took chest X-ray at the bedside, and the inflammatory indicators were redetected. However, the results of Chest X-ray and the inflammatory indicators showed no signs of acute pulmonary infection or pulmonary tuberculosis. Therefore, the hypothesis that the significant increase of CRP and ESR was due to pulmonary infection was excluded. At the same time, male tumor markers and CT examination showed no obvious abnormality, so the increase of CRP and ESR caused by tumor was also excluded.

**Figure 3:**
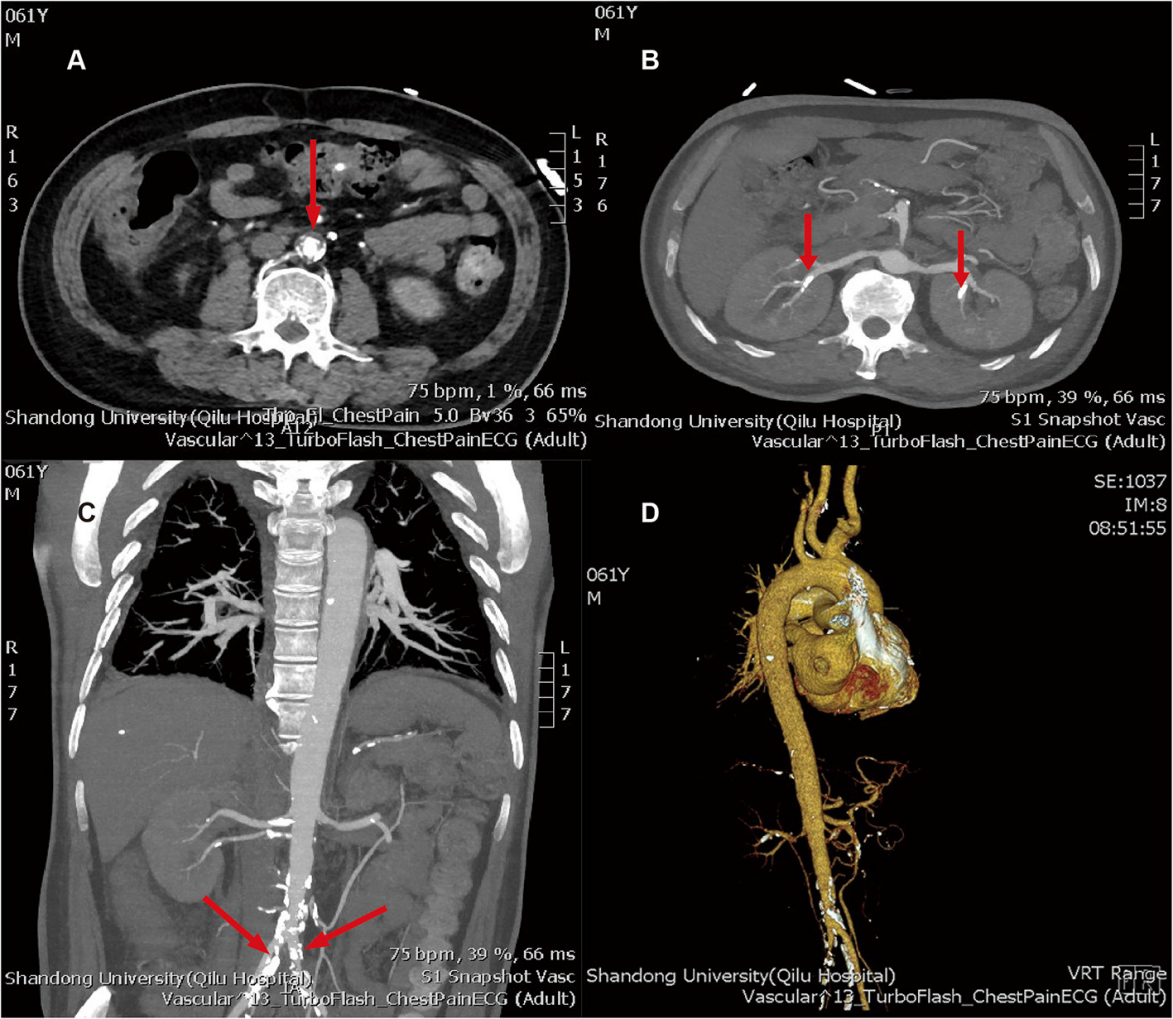
**(A)** aortic atherosclerosis; **(B)** bilateral renal arteriosclerosis and multiple stenosis; **(C)** multiple atherosclerosis and lumen stenosis of bilateral iliac arteries; **(D)** three dimensional reconstructions of artery. The arrows above refer to the stenosis of the artery.

**Figure 4:**
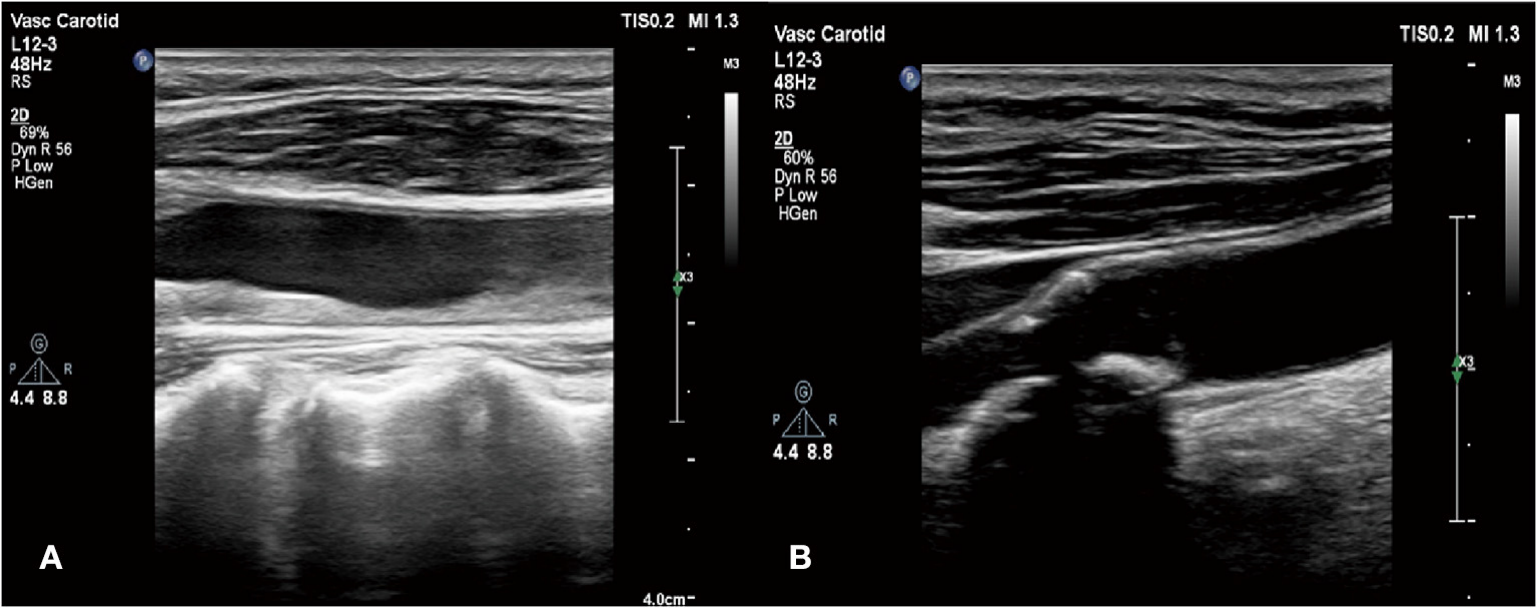
**(A)** Stenosis in the initial segment of left internal carotid artery and left external carotid artery; **(B)** stenosis in the right external carotid artery.

Based on this, we conducted a careful physical examination of the patients. Palpation of bilateral radial artery showed that the pulsation of bilateral radial artery was inconsistent, the pulsation of dorsalis pedis artery was not touched, and vascular murmur could be heard by auscultation of carotid artery. Further detection of the blood pressure of the limbs: the left upper limb was 137/64 mmHg, the right upper limb was 132/52 mmHg, the left lower limb was 90/23 mmHg, and the right lower limb was 113/28 mmHg. We can find that the lower limb blood pressure is significantly lower than the upper limb blood pressure. According to the results of blood pressure and physical examination of limbs, and the increase of ESR, CRP, and other inflammatory indicators, we considered that the patient may be TA. Therefore, we improved CT angiography (CTA) examination; the results showed aortic atherosclerosis and multiple stenosis of its branches, and some showed moderate-to-severe stenosis, bilateral renal arteriosclerosis and multiple stenosis (Figure 3). And the results of carotid artery ultrasound showed stenosis in the initial segment of left internal carotid artery, left external carotid artery, and right external carotid artery (Figure 4).

We asked rheumatologists for consultation. According to the 1990 American College of Rheumatology (ACR) criteria, the consultation opinion was TA, so we added the diagnosis of TA. The ACR criteria for TA consist of (1) age of the onset before 40 years old; (2) claudication of an extremity; (3) decreased brachial artery pulse; (4) a difference of more than 10-mmHg systolic pressure between two limbs; (5) a bruit over subclavian arteries or the aorta; and (6) angiographic evidence of narrowing or occlusion of the aorta, its primary branches, or large arteries in the proximal upper or lower extremities. If three of the above six items are met, it can be diagnosed as TA (3). Intravenous dexamethasone 10 mg daily, calcium carbonate D3, calcitriol capsules, and other oral drug treatments were tailored to treat TA. After 4 days of treatment, Hs-CRP was decreased to 11.67 mg/L, and ESR was decreased to 91. mm/h. The inflammatory index was significantly descended, and NT-pro BNP decreased to 3,100 pg/ml.

## Discussion

Takayasu arteritis is a rare chronic granulomatous inflammation involving the aorta and its main branches. In 1908, Takayasu, a Japanese ophthalmologist, first discovered and described TA (4). According to statistics, the incidence rate of the disease is 2/10,000 per year (5). Most adult cases are female, with an incidence rate of 90%, and the age of the onset is relatively small, usually between 20 and 40 years (6). Another study found that the incidence rate of TA in children showed different male-to-female ratios (7, 8). A Japanese study showed that among newly diagnosed patients with TA, although male patients were significantly less than female patients, the median age of the onset of male patients was significantly higher than that of female patients, and male patients usually had extensive aortic lesions or aneurysms and more complications (9).

The main pathological manifestations of TA are irregular thickening of aortic wall, proliferation of endothelial cells, fibrosis, and contraction of media and adventitia, accompanied by various types of inflammation, such as acute exudative inflammation, chronic non-specific inflammation, and various granulomatous inflammations (10). The pathological manifestations of different stages are different: the early inflammatory process involves all layers of the vascular wall, showing the accumulation of inflammatory cells, such as lymphocytes (11, 12), the middle stage shows the intimal hyperplasia of the arterial wall and the progress of the inflammatory process of the media and adventitia, and the end stage shows the diffuse or nodular fibrosis of perivascular tissue, followed by narrowing of the lumen (13–15). When the rapid progression of the disease leads to insufficient and delayed fibrosis, the vascular wall may become thinner and lead to aneurysmal dilatation (16). This may be the mechanism of TA-induced coronary artery stenosis.

As its name suggests, TA mainly involves the aorta and its main branches. The lesion also affects the coronary artery. When the coronary artery is involved, it is usually caused by the direct expansion of the aortic wall inflammation, leading to the proximal stenosis of the coronary artery. It has been reported that the incidence of TA involving coronary artery is about 10%, and the lesion can be divided into three main types: type 1, coronary artery stenosis or proximal coronary artery stenosis; type 2, diffuse or focal coronary arteritis; type 3, coronary aneurysm (17). Type 1 was the most common type (18).

Eleven years ago, the patient had undergone coronary angiography because of acute myocardial infarction. The results showed multiple coronary artery stenoses. After evaluation, CABG was performed in our hospital. According to the results of coronary angiography in that year, the coronary artery lesions of the patient were in accordance with the above Type 1. Acute myocardial infarction occurred again 11 years later, and coronary angiography showed restenosis of the previous stenotic coronary artery branch. It has been reported that the main problem of the patients with TA, receiving CABG, is graft restenosis. In addition to poor control of the blood lipid level, we considered the influence of autoimmune disease, namely, TA, according to the physical signs of the patient, elevated inflammatory indexes, and the results of CTA. Of course, we should rule out the possibility of other vascular diseases. Congenital coarctation of aorta has no inflammatory activity, and the stenosis is limited to the junction of arterial duct, which can be distinguished from Ta; in addition, it needs to be distinguished from coronary artery stenosis caused by atherosclerosis. The LDL-C level of the patient is normal, so atherosclerosis can be ruled out. Finally, Ta needs to be distinguished from thromboangiitis obliterans (Buerger disease), which often occurs in small- and medium-sized arteries and veins of the limbs. We tested the gene of the patient and found that there was a heterozygous variation at the CD36 susceptibility-related gene. Yagi et al. mentioned in the article that there is a certain link between TA and CD36 deficiency (19). Due to genetic susceptibility, this also explains the cause of acute myocardial infarction caused by TA involving the coronary artery again after CABG. In addition, according to the epidemiology of TA, we found that the disease mainly occurred in young women, while our patient was a 61-year-old man, which should be paid enough attention by clinicians. Some pieces of literature have shown that glucocorticoid pretreatment intervention to inhibit active inflammation has a good prognosis, the 5-year survival rate is 87%, the 10-year survival rate is 81% (20). Therefore, after definite diagnosis, we gave the patient dexamethasone treatment to control the activity of inflammation. With the treatment, inflammatory indexes decreased and activity was inhibited. For further treatment, although the patient still had multibranch stenosis of coronary artery, considering previous CABG operation history of the patient, surgery and interventional therapy were not feasible; the patient was given conservative drug treatment.

## Conclusions

Therefore, we get the following facts from this case: When patients with acute myocardial infarction, especially patients after CABG, have typical precordial pain symptoms, in addition to considering the risk factors, such as age, gender, hypertension, and blood lipid level, we should actively explore other diseases other than the main diagnosis, such as coronary artery involvement caused by TA.

## Data Availability Statement

The original contributions presented in the study are included in the article, further inquiries can be directed to the corresponding author/s.

## Ethics Statement

The studies involving human participants were reviewed and approved by the Ethics Committee of the Qilu Hospital of Shandong University. The patient provided their written informed consent to participate in this study. Written informed consent was obtained from the patient for publication of this case report and any accompanying images.

## Author Contributions

HY, WL, and YZ wrote the report. XY and NL took the pictures. YT and PB performed the research and revised the report. All authors have read and approved the final version of the manuscript.

## Funding

This work was supported by the State Key Program of National Natural Science Foundation of China [Grant No. 81530014], National Key R&D Plan of China [Grant No. 2017YFC1700502], the Natural Science Foundation of Shandong Province [Grant No. ZR2019QH010], Cardiovascular Multidisciplinary Integrated Research Fund [Grant No. z-2016-23-2101-10], and the program of educational reform and research project of Cheeloo College of Medicine, Shandong University [Grant No. qlyxjy-202037]. The funders had no role in the study design, data collection and analysis, and the decision to publish or the preparation of the manuscript.

## Conflict of Interest

The authors declare that the research was conducted in the absence of any commercial or financial relationships that could be construed as a potential conflict of interest.

## Publisher's Note

All claims expressed in this article are solely those of the authors and do not necessarily represent those of their affiliated organizations, or those of the publisher, the editors and the reviewers. Any product that may be evaluated in this article, or claim that may be made by its manufacturer, is not guaranteed or endorsed by the publisher.
